# Molecular basis of host-adaptation interactions between influenza virus polymerase PB2 subunit and ANP32A

**DOI:** 10.1038/s41467-020-17407-x

**Published:** 2020-07-21

**Authors:** Aldo R. Camacho-Zarco, Sissy Kalayil, Damien Maurin, Nicola Salvi, Elise Delaforge, Sigrid Milles, Malene Ringkjøbing Jensen, Darren J. Hart, Stephen Cusack, Martin Blackledge

**Affiliations:** 1 Univ. Grenoble Alpes, CNRS, CEA, IBS, F-38000 Grenoble, France; 2 European Molecular Biology Laboratory - EMBL, 71, Avenue des Martyrs, Grenoble, France

**Keywords:** Intrinsically disordered proteins, Influenza virus, Solution-state NMR

## Abstract

Avian influenza polymerase undergoes host adaptation in order to efficiently replicate in human cells. Adaptive mutants are localised on the C-terminal (627-NLS) domains of the PB2 subunit. In particular, mutation of PB2 residue 627 from E to K rescues polymerase activity in mammalian cells. A host transcription regulator ANP32A, comprising a long C-terminal intrinsically disordered domain (IDD), is responsible for this adaptation. Human ANP32A IDD lacks a 33 residue insertion compared to avian ANP32A, and this deletion restricts avian influenza polymerase activity. We used NMR to determine conformational ensembles of E627 and K627 forms of 627-NLS of PB2 in complex with avian and human ANP32A. Human ANP32A IDD transiently binds to the 627 domain, exploiting multivalency to maximise affinity. E627 interrupts the polyvalency of the interaction, an effect compensated by an avian-unique motif in the IDD. The observed binding mode is maintained in the context of heterotrimeric influenza polymerase, placing ANP32A in the immediate vicinity of known host-adaptive PB2 mutants.

## Abstract (web summary)

Avian influenza polymerase undergoes host adaptation in order to efficiently replicate in human cells. Here, the authors use NMR spectroscopy and quantitative ensemble modelling to describe the highly dynamic assemblies formed by the human-adapted or avian-adapted C-terminal domains with the respective ANP32A host proteins.

## Introduction

Influenza A virus (IAV) is responsible for 3–5 million severe cases every year, resulting in 250–500,000 deaths^1^. Most influenza strains evolve exclusively in the large reservoir of water birds, but some highly pathogenic avian strains (e.g., H5N1, H5N8 and H7N9) can infect humans with lethal consequences (up to 60% mortality) and are potential pandemic threats for humanity if they develop human-to-human transmissability^2^. However, for these avian *(av)* viruses to efficiently replicate in mammalian cells, host adaptation of the viral polymerase is necessary. Replication of IAV is carried out by the RNA-dependent RNA viral polymerase that functions as a heterotrimeric complex, formed from separate components PA, PB1 and PB2. Few mutations are required for avian to human adaptation^3–6^, and a number of these cluster on the surface of the C-terminal 221 amino acid section of PB2, comprising separate ‘627’ and ‘NLS’ domains^4,7^. In particular mutation of residue 627 from E to K in *av*PB2 rescues polymerase activity and viral replication in mammalian cells^8–11^. Members of the host transcription regulator family ANP32^12^, comprising a long low-complexity acidic intrinsically disordered domain (IDD; sometimes known as LCAR) at the C-terminus, have been shown to be responsible for this viral adaptation^13^. Human ANP32A (*h*ANP32A) lacks an insertion of 33 disordered residues compared to *av*ANP32A, restricting *av*H5N1 polymerase activity in mammalian cells. This restriction is lifted by E627K mutation, suggesting an essential role for ANP32A through interaction with PB2^14–24^, although there are currently no molecular descriptions of these interactions. The interaction between members of the ANP32 family and influenza polymerase is critical in supporting IAV replication, and is attracting increasingly intense interest^16,18,19^. Recent studies also demonstrate that the interaction occurs in the nucleus^24^, and further studies point to the importance of related members of the ANP32 family, in particular ANP32B^20–22^, as well as the role of surface residues in the folded leucine-rich region (LRR) of ANP32A^23^.

Conformationally, the 627-NLS region of PB2 exhibits intriguing behaviour^7,25^. X-ray crystallographic structures of both *h* and *av-*adapted 627-NLS revealed a compact two-domain structure^4,7^, a conformation also found in full-length transcriptionally active polymerase^26^. Solution NMR, however, revealed the coexistence of two forms of 627-NLS, corresponding to ‘open’ and ‘closed’ states that interchange in a highly dynamic equilibrium^27^, while crystallographic investigation of the transcriptionally inactive polymerase complex suggested a role for this open form in viral replication or polymerase assembly^28,29^.

Here, we combine NMR and quantitative ensemble analysis to describe and compare the complexes formed between *av*ANP32A and a*v-*adapted 627-NLS (627-NLS(E)), and between *h*ANP32A and *h*-adapted 627-NLS (627-NLS(K)). Although the complexes are both found to be highly dynamic they exhibit significant differences. Polyvalent combinations of transient interactions between the acidic IDD and the positively charged 627(K) domain stabilise the *h*ANP32A:627-NLS(K) complex. This polyvalency is less efficient for *av*ANP32A:627-NLS(E), due to the interruption of an exposed basic surface on the 627 domain by the presence of E627. The weaker interaction is however compensated by the recruitment of additional sequences on the longer *av*IDD, in particular an avian-specific hexapeptide motif that interacts with the linker between 627 and NLS. Notably the cross-interaction between *h*ANP32A and 627-NLS(E) exhibits neither of these possible stabilisation mechanisms, which may be related to the inability of IAV polymerase to function in human cells without the E627K mutation. Importantly, we show that the interaction exhibits the same properties in the presence of heterotrimeric influenza polymerase, providing insight into the role of the IDD in the function of putative ANP32A:polymerase complexes.

## Results

### IDDs of *h* and *av*ANP32A and their interactions with 627-NLS

We have compared the two complexes using solution state NMR, initially from the side of ANP32A. The IDDs of *h* and *av*ANP32A comprise 63/96 and 79/129 Asp or Glu residues, respectively, leading to extensive spectral overlap. *h* and *av*ANP32A IDDs differ principally due to a 33 amino acid insert in *av*ANP32A (176–209) comprising an *av*-unique hexapeptide, ^176^VLSLVK^181^, followed by a duplication of 27 amino acids also present in *h*ANP32A IDD. Backbone resonance assignment was completed to 78% and 58%, respectively, revealing that *h* and *av*ANP32A IDDs are indeed both intrinsically disordered (Supplementary Figs. 1 and 2), with a slight tendency (20%) towards helical conformation for the hexapeptide, but negligible secondary structural tendency elsewhere.

**Fig. 1:**
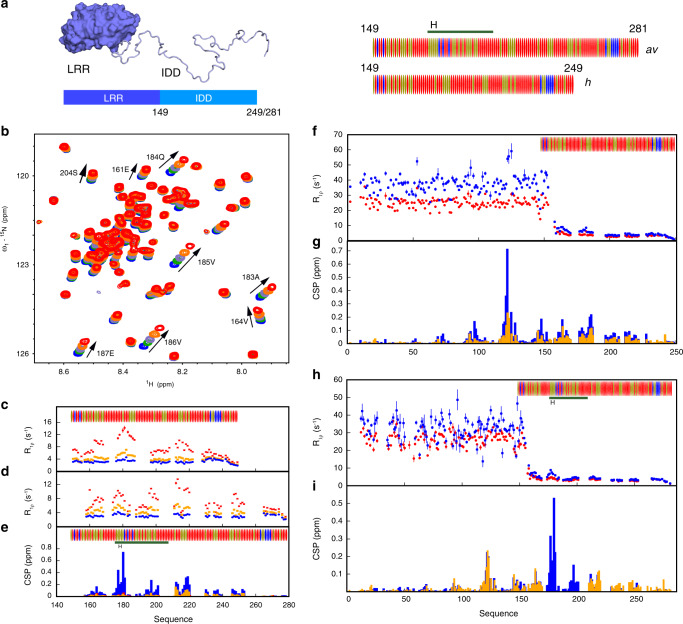
NMR of ANP32A with 627-NLS reveals a highly dynamic polyvalent complex. **a** Representation of the domains of ANP32A. Blue surface shows the LRR domain, pale blue ribbon-disordered IDD (100 or 132 amino acids). Sequence distribution of IDDs shown on right. Red = acidic, blue = basic and beige = hydrophobic or polar. *Av*IDD contains a 33 amino acids insert (horizontal line) comprising an avian-unique hexapeptide (H). **b** Chemical shift titration of 627(K) into ^15^N-labelled IDD of *h*ANP32A. *h*ANP32A was 25 μM throughout. 627(K) concentrations were 0 (blue), 25 (green), 50 (grey-blue), 100 (orange) and 200 (red) μM. Measurement at 850 MHz, 293 K (see ‘Methods’ section). **c** ^15^N rotating frame relaxation (*R*_1ρ_) of free (blue) ^15^N-labelled IDD of *h*ANP32A at 300 μM, and upon mixing with 627(K) at 1:1 (orange) and 1:4 (red) ratio. Measurement at 850 MHz, 293 K. **d** ^15^N rotating frame relaxation (*R*_1ρ_) of free (blue) ^15^N-labelled IDD of *av*ANP32A at 300 μM, and upon mixing with 627(E) at 1:1 (orange) and 1:4 (red) ratio. Maximum effect is seen at the equivalent motif, 33 amino acids downstream, from *h*ANP32A. **e** Chemical shift perturbation (CSP) of full-length *av*ANP32A upon addition of 627(E) (orange), NLS (red) and 627-NLS(E) (blue). Concentration of *av*ANP32A was 25 μM and the partner 50 μM throughout. Resonances from the LRR were not observable at this low concentration. **f** ^15^N rotating frame relaxation (R_1ρ_) of free (red) ^15^N-labelled full-length *h*ANP32A at 300 μM, and upon mixing with 627-NLS(K) at 1:1 ratio (blue). Measurement at 850 MHz, 293 K. **g** CSP of full-length *h*ANP32A upon addition of 627-NLS(K) (blue). Concentration of *h*ANP32A was 300 μM and 627-NLS(K) 600 μM. The cross-interaction between *h*ANP32A and 627-NLS(E) is shown for comparison at the same stoichiometric ratio (orange). **h** ^15^N rotating frame relaxation (*R*_1ρ_) of free (red) ^15^N-labelled full-length *av*ANP32A at 300 μM, and upon mixing with 627-NLS(E) at 1:1 ratio (blue). Measurement at 850 MHz, 293 K. **i** CSP of full-length *av*ANP32A upon addition of 627-NLS(E) (blue). Concentration of *av*ANP32A was 300 μM and 627-NLS(E) 600 μM. The cross-interaction between *h*ANP32A and 627-NLS(E) is shown for comparison at the same stoichiometric ratio (orange). The avian-specific insert is highlighted by shifting data from *h*ANP32A from residues following the beginning of the insert by 33 amino acids.

Upon addition of the 627(K) domain, ^1^H and ^15^N chemical shift perturbations (CSPs) are seen for a large number of resonances in the IDD of *h*ANP32A (Fig. 1b, Supplementary Fig. 1d). NMR relaxation rates measured at increasing titration admixtures (Fig. 1c) show maximal effects for the acidic strand ^180^DEDA^183^, while the largest CSPs are seen for the adjacent hydrophobic residues ^184^QVV^186^ (Fig. 1b, Supplementary Fig. 1d). Additional interactions are seen throughout the chain, in particular at ^164^VE^165^ and ^214^YND^216^. Comparison of backbone ^13^C shifts in the free and fully bound states reveals that only ^179^YDED^182^ shows any evidence of folding upon binding (Supplementary Fig. 1d), in this case into an extended β-sheet conformation, while the remainder of the chain remains highly flexible in the complex, retaining its random coil nature (Supplementary Fig. 1b). Similar evidence of multiple interaction sites is seen for the IDD of *av*ANP32A in complex with the 627(E) domain (Supplementary Fig. 1d, Fig. 1d). Relaxation properties (Fig. 1c, h) and more specifically CSPs (Fig. 1e) of the hexapeptide are strongly influenced by the presence of both domains of 627-NLS, compared to constructs each comprising only the 627 or NLS domains and the linker. This implies that the specific conformational behaviour of the linker in integral 627-NLS, or the relative position of the two domains, are essential for the interaction with the hexapeptide of *av*ANP32A IDD.

Interaction of 627-NLS and ANP32A reveals similar profiles to those measured for the IDDs alone, with additional interactions involving the LRR for both *h* and *av*ANP32A (Fig. 1f–i). In both *h* and *av*ANP32A, the largest CSPs in the LRR are centred on ^120^LFN^122^, with additional shifts induced following the spine of the beta-helix (Fig. 1g). Notably, the cross-interaction, between *h*ANP32A and 627-NLS(E) shows much smaller shifts than *h*ANP32A:627-NLS(K), while comparison with *av*ANP32A:627-NLS(E) clearly identifies increased shifts centred on the hexapeptide (Fig. 1i).

Although most of the IDD is involved in the interaction, the strongest binding or highest populations of binding interactions, occur at a distance of 25–35 amino acids from the LRR. In the case of *av*ANP32A, this concerns the avian-unique hydrophobic hexapeptide, and in *h*ANP32A the sequence ^180^DEDAQVV^186^. Further interactions are observed downstream of these interaction sites until the nuclear localisation sequence (KRKR), situated ~15 amino acids from the end of the chain, beyond that point no significant interactions are observed.

Despite the evidence of clear interaction between ANP32A and 627-NLS, the complex is polyvalent, and in all cases weak (Supplementary Table 1, Supplementary Fig. 3), with none of the individual interactions exhibiting a stronger affinity than 800 μM for the interaction of *h*ANP32A with 627(K) (1700 μM for *av*ANP32A with 627(E)). Notably, the interactions between *h*ANP32A and 627(E) are also weaker (>1400 μM), suggesting that the single E627K mutation plays an important role. Interaction with integral 627-NLS is weaker due to the open-closed equilibrium reducing the population of available binding states. Interaction of the LRR of ANP32A alone with the 627 domain reveals weaker affinity (>1500 μM). Although these interactions are weak, the extended interaction surface involving 80–100 disordered amino acids in *h* and *av*ANP32A nevertheless results in tighter binding as experienced by 627-NLS.

### Interactions of *av* and *h*-adapted 627-NLS with *av* and *h*ANP32A

**Fig. 2:**
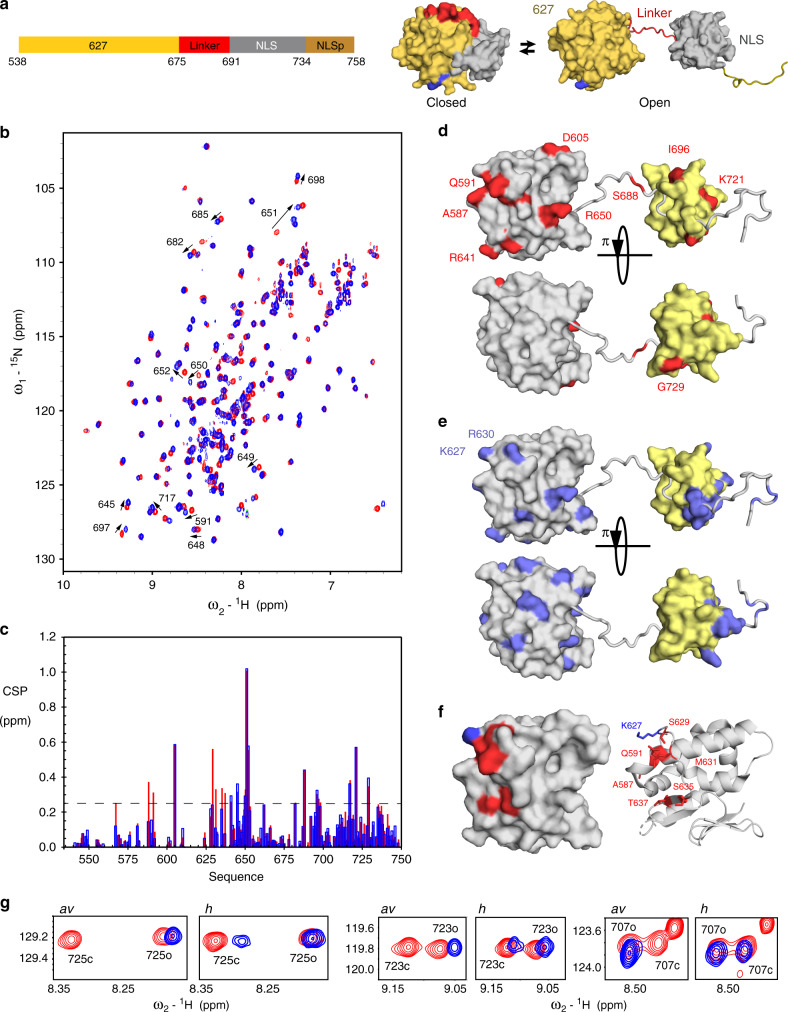
Interaction of 627-NLS and ANP32A observed from the perspective of 627-NLS. **a** Representation of the domains of 627-NLS. The C-terminal domains of PB2 comprise two sub-domains (627—orange and NLS—grey) connected by a linker (red) and terminated by an NLS peptide (NLS). The molecule exists in equilibrium between closed and open forms that exchange at 50 s^−1^ at room temperature. The position of the E627K adative mutation is shown in blue. **b** HSQC of the open-only form of ^2^D, ^13^C and ^15^N-labelled 627-NLS(K) (D730A/E687A; 200 μM; red), indicating chemical shifts of selected sites upon addition of *h*ANP32A IDD (400 μM blue). **c** CSP of 627-NLS(K) (300 μM) upon addition of *h*ANP32A IDD at a ratio of 1:4 (red) and 627-NLS(E) (300 μM) upon addition of *av*ANP32A IDD at a ratio of 1:4 (blue). Spectra recorded on ^2^D, ^13^C and ^15^N-labelled 627-NLS at 293 K and 850 MHz. Only shifts affecting resonances corresponding to the open form are shown for clarity. **d** CSP of 627-NLS(K) (red) induced by addition of *h*ANP32A derived from **b** above the threshold of 0.25 (dashed line) for 627 (grey) and NLS (yellow) domains. Two orientations of the protein are shown. **e** Distribution of basic sidechains (blue) on the surface of the 627 and NLS domains. By comparison with **c**, it is evident that one of the two faces preferentially interacts with the IDD. **f** Represention of the largest differential shifts on the surface of the 627 domain upon addition of ANP32A as shown in **c**. Amino acids showing the largest difference in CSP (>0.15) in the 627-NLS(K):*h*ANP32A IDD complex compared to the 627-NLS(E):*av*ANP32A IDD are highlighted in red (position of 627 shown in blue). **g** Interaction of full-length 627-NLS with ANP32A induces a change in the open-closed equilibrium. Red—peaks reporting on the open and closed forms of 627-NLS(E) or 627-NLS(K) before the addition (blue) of *h* or *av*ANP32A, respectively. The interaction with ANP32A potentiates the equilibrium in both cases, fully removing the closed form in the case of the avian pair. Residues distal from the main interaction site were chosen to reduce the risk of peak disappearance due to direct interaction.

The interaction was also investigated from the side of 627-NLS domains of PB2. The two domains exhibit an open-closed equilibrium (Fig. 2a) that is populated ~40:60 at 293 K leading to two sets of resonances for the majority of the protein^27^. Addition of *h*ANP32A to 627-NLS(K) resulted in CSPs throughout the protein (Supplementary Fig. 4). The largest shifts are observed in the open form, suggesting that ANP32A interacts preferentially with this conformation. The closed form of 627-NLS(K) is stabilised by a tripartite salt bridge, and can be removed from the equilibrium by mutation of the implicated amino acids (R650 or D730/E687)^27^. CSPs induced upon interaction with the ANP32A IDD are illustrated for clarity using an open-only mutant (Fig. 2b), and the distribution of CSPs as measured on the wild-type proteins (Fig. 2c). Figure 2d illustrates the major shifts observed for 627-NLS(K) upon addition of *h*ANP32A IDD. Although basic sidechains are found on both sides of the domain (Fig. 2e), CSPs are observed mainly on one face of the 627 domain, again suggesting that the interaction is not uniquely driven by electrostatic attraction with the acidic IDD. Despite strong similarity of the CSP profiles, clear additional shifts are measurable for 627-NLS(K):*h*ANP32A compared to 627-NLS(E):*av*ANP32A (Fig. 2c, Supplementary Fig. 4), particularly in the vicinity of R630, L636 and 587–591, forming a continuous interaction surface that is strongly enhanced in 627-NLS(K) (Fig. 2f). It is interesting to note that residues 590 and 591 provide an alternative pathway to host adaptation, as evidenced in the 2009 pandemic strain^5,30^. The long extended loop comprising 627E/K, that is highly flexible in both 627E and 627 K (Supplementary Fig. 5), wraps around the 588–605 helix and interaction with *h*ANP32A appears to enhance the accessibility of the N-terminus of this helix to the ANP32A IDD uniquely when 627 K is present.

The affinity measured when observing resonances from 627-NLS(K) is considerably tighter than from the side of *h*ANP32A, with values of 20 μM for 627(K) and 50 μM for 627-NLS(K). This increased affinity apparently occurs due to avidity with the extensive interaction surface presented by *h*ANP32A. Although the stoichiometry cannot be determined accurately, titration curves imply that it is significantly different to 1:1 from the side of 627-NLS (Supplementary Fig. 3), again suggesting that the increased affinity occurs due to polyvalent binding of multiple, weak binding sites on ANP32A to each site on the surface of 627-NLS(K). Notably, the affinity for *av*ANP32A IDD measured when observing resonances of 627(E) is ~20 times weaker than for the equivalent human IDD:627(K) complex, with values >600 μM. In combination with observations measured from the side of ANP32A, it therefore appears that the absence of K627, which disrupts the continuity of the positively charged surface on 627, strongly abrogates this component of the interaction.

In addition, the interaction with ANP32A strongly favours the open form of 627-NLS (Fig. 2g), especially for the avian interaction, with the closed form essentially disappearing from the equilibrium at 1:2 ratio of 627-NLS(E):*av*ANP32A and falling from 65 to 40% at the same stoichiometry in 627(K)-NLS:*h*ANP32A.

The polyvalent nature of the interaction of the IDD with 627-NLS is further substantiated by the comparison of the CSP-derived profiles measured when individual adjacent peptides from *hu*ANP32A IDD interact with the open-mutant of 627-NLS(K) (Supplementary Fig. 6). These results demonstrate that both peptides interact with residues on the surface of 627-NLS, some that are common to both peptides and to the full-length IDD (Fig. 2b), and others that are unique to one or other of the peptides, confirming the multivalent, transient and dynamic nature of the interaction of the full-length *hu*ANP32A IDD with 627-NLS(K). One of these peptides, that comprises 11 consecutive acidic amino acids, also binds much tighter to the open form of 627-NLS(K) than to the open form of 627-NLS(E) (Supplementary Fig. 7), again supporting the observation that the contribution of K627 to the positively charged surface of the 627(K) domain is the essential factor for the tighter interaction with the negatively charged regions of *hu*ANP32A IDD. These data therefore further support our model of differentiation of the binding modes of the two complexes.

### Ensemble descriptions of dynamic ANP32A:627-NLS complexes

**Fig. 3:**
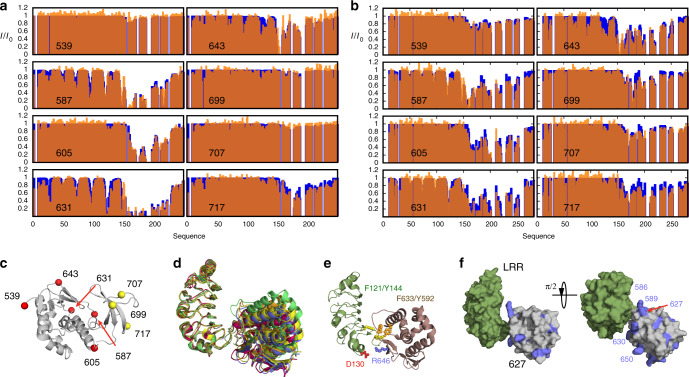
Experimental characterisation of the dynamic 627-NLS:ANP32A interaction complexes. **a** Experimental (orange) and calculated (blue) paramagnetic relaxation enhancements measured on *h*ANP32A in the presence of paramagnetically labelled 627-NLS. Intensity ratios compare spectra recorded in the presence of oxidised and reduced forms of TEMPO-maleimide for complex admixtures of *h*ANP32A (300 μM) and 627-NLS(K) (150 μM). Calculated values result from representative ensembles selected using the ASTEROIDS approach. **b** Experimental paramagnetic relaxation enhancements measured on *av*ANP32A in the presence of paramagnetically labelled 627-NLS. Intensity ratios compare spectra recorded in the presence of oxidised and reduced forms of TEMPO-maleimide for complex admixtures of *av*ANP32A (220 μM) and *av*627-NLS (400 μM). This admixture was chosen to replicate the population of bound state estimated in the case of 627-NLS(K):*h*ANP32A. **c** Position of the eight cysteine mutations used to label 627-NLS. Five mutations were selected over the surface of the 627 domain and three on NLS. One mutant protein was expressed and purified for each site on both 627-NLS(K) and 627-NLS(E), and labelled with TEMPO-maleimide (see ‘Methods’ section). **d** Optimisation of the position of the folded domains of *h*ANP32A and 627-NLS(K). Two thousand starting poses generated by the programme Haddock were optimised with respect to experimental PREs measured on the folded domain. The ten best fitting poses are shown. The same procedure was used for PREs measured on the *av*ANP32A: 627-NLS(E) complex, resulting in similar best fitting structures. **e** Localisation of intermolecular interactions possibly stabilising the interface between the two proteins, involving hydrophobic (F121 and Y144 in ANP32A, and Y592 and F633 in 627-NLS) and electrostatic interactions (D130 in ANP32A and R646 in 627-NLS). **f** Position of positively charged ridge of solvent-exposed basic sidechains in the vicinity of the interface between the folded domains.

To develop a more detailed description of the dynamic interaction between 627-NLS and ANP32A, we have incorporated eight cysteine mutants into both 627-NLS(K) and (E), and individually labelled the proteins with single TEMPO-based paramagnetic spin-label. This allows the detection of paramagnetic relaxation enhancements (PREs), from the perspective of five positions on the 627 domain and three on the NLS domain (Fig. 3a–c). PREs report on weak, or sparsely populated contacts between the two proteins, providing long-range positional constraints that are complementary to the short-range modulation of the electronic and chemical environment probed by CSPs, and the modulation of the dynamics of each site as measured by spin relaxation. These orthogonal experimental probes reveal different aspects of the same interfaces, while providing a high level of confirmatory experimental validation (vide infra).

The experimental results show strong PREs, reporting on tighter or more populated interactions, distributed over long stretches of the IDD for certain spin-label positions, and little broadening for other positions. Such profiles again suggest a polyvalent interaction, whereby distinct sites dispersed along the IDD visit the same sites on 627-NLS. Interestingly, some labels (in particular 587 and 631) induce a well-defined pattern over the long β-sheet on the concave face of the LRR of ANP32A, allowing the determination of its orientation with respect to the 627 domain (Fig. 3d, see ‘Methods’ section), despite the weak interaction between the two domains (Supplementary Table 1). This orientation is in full agreement with the observed CSP on the surface of the LRR of ANP32A (Fig. 1), where the largest chemical shifts are seen for F121, Y122 and C123. These residues are positioned in closest proximity to the 627 domain in the ensemble of conformers that best fit the entire set of experimental data. The interaction is apparently stabilised by hydrophobic interactions, involving residues on the surface of 627 and ANP32A, and an electrostatic interaction between D130 (ANP32A) and R646 (627) (Fig. 3e). It seems likely that the observed interaction relates to observations that have recently implicated D130 in host adaptation^20,21,23^. The optimal poses determined for the 627-NLS(K):*h*ANP32A and 627-NLS(E):*av*ANP32A complexes are very similar. The interface between 627 and ANP32A is bordered by a nearly continuous ridge of solvent accessible basic sidechains, including K589, R630, R641 and R650, that is completed by the presence of K627 in the case of 627-NLS(K) (Fig. 3f). Inspection of the PRE data from the IDD regions of *h* and *av*ANP32A reveals weaker effects in the region immediately following the LRR for *av*ANP32A, and more contacts with the dislocated NLS domain (Fig. 3a, b), in particular the spin-label at position 699 in the NLS broadens the IDD maximally at the position of the hexapeptide of *av*ANP32A. This again confirms that the interaction with the immediately proximal basic face of 627, defined by the differential CSPs (Fig. 2f), is weaker in the case of *av*ANP32A.

**Fig. 4:**
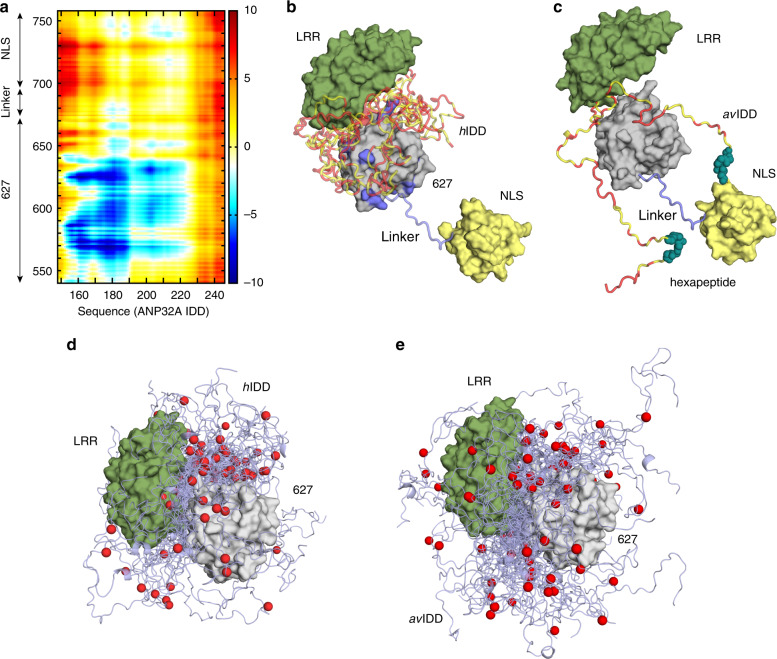
Comparison of 627-NLS(K):*h*ANP32A and 627-NLS(E):*av*ANP32A interaction complexes. **a** Average distance difference matrix, showing the average difference (*d*_*hh*_ − *d*_*avav*_) in distance between amino acid positions in the IDD of ANP32A (*x*-axis) and the 627-NLS domains (*y*-axis) over the two ensembles. The colour code shown on the right is measured in Å. **b** Representation of the key interactions between *h*ANP32A and 627-NLS(K). Polyvalent interactions between 627 and the IDD localise the disordered domain in the vicinity of the basic patch on the surface of 627 (see Fig. 3). The different IDD chains are shown to represent different binding modes and are truncated at residue 200 for clarity. Red positions in the IDD indicate the acidic sidechains. Note that this representation illustrates the tendency over the entire ensemble that is highly disperse (see **d** and **e**). **c** Representation of the key interactions between *av*ANP32A and 627-NLS(E). In this case, the average distance between the IDD and the surface of 627 is larger, but in general closer to the NLS domain, in particular the hexapaptide of ANP32A, and the linker between 627 and NLS. The two IDD chains represent reduced polyvalency compared to *h*ANP32A and 627-NLS(K), and are again truncated at residue 200 for clarity. **d** Representative ensemble of conformations describing the conformational space sampled by the *h*ANP32A:627-NLS(K) complex. The NLS domain has been removed for clarity, and the IDD truncated at residue 210. The position of residue 190 is indicated as a red sphere. **e** Representative ensemble of conformations describing the conformational space sampled by the *av*ANP32A:627-NLS(E) complex. Representation as in **d**.

Ensemble analysis of the PRE data using the ASTEROIDS approach^31^, accounting for the flexibility in the 627-NLS linker and IDD domains of ANP32A (Supplementary Fig. 8), allows us to propose a molecular description of the conformational sampling of the entire complex. Representative ensembles of conformations of the multi-domain complex (Fig. 4) reveal the sampling of conformational space of the IDD and NLS domains relative to the position of 627 and the LRR of ANP32A. Comparison of the position of the IDDs over the ensembles (Fig. 4d, e) reveals more restricted sampling for *h*ANP32A, localising residues 175–200 in the vicinity of the basic ridge on the surface of 627. Sampling of *av*ANP32A is more dispersed (Fig. 4b–e), as shown quantitatively in the average distance map (Fig. 4a), that identifies closer contacts in the 627-NLS(K):*h*ANP32A complex for residues 160, 180 and 205 with 570, 586 and 627, and closer contacts between the linker and NLS regions, and *av*ANP32A region 150–200 (Fig. 4a).

**Fig. 5:**
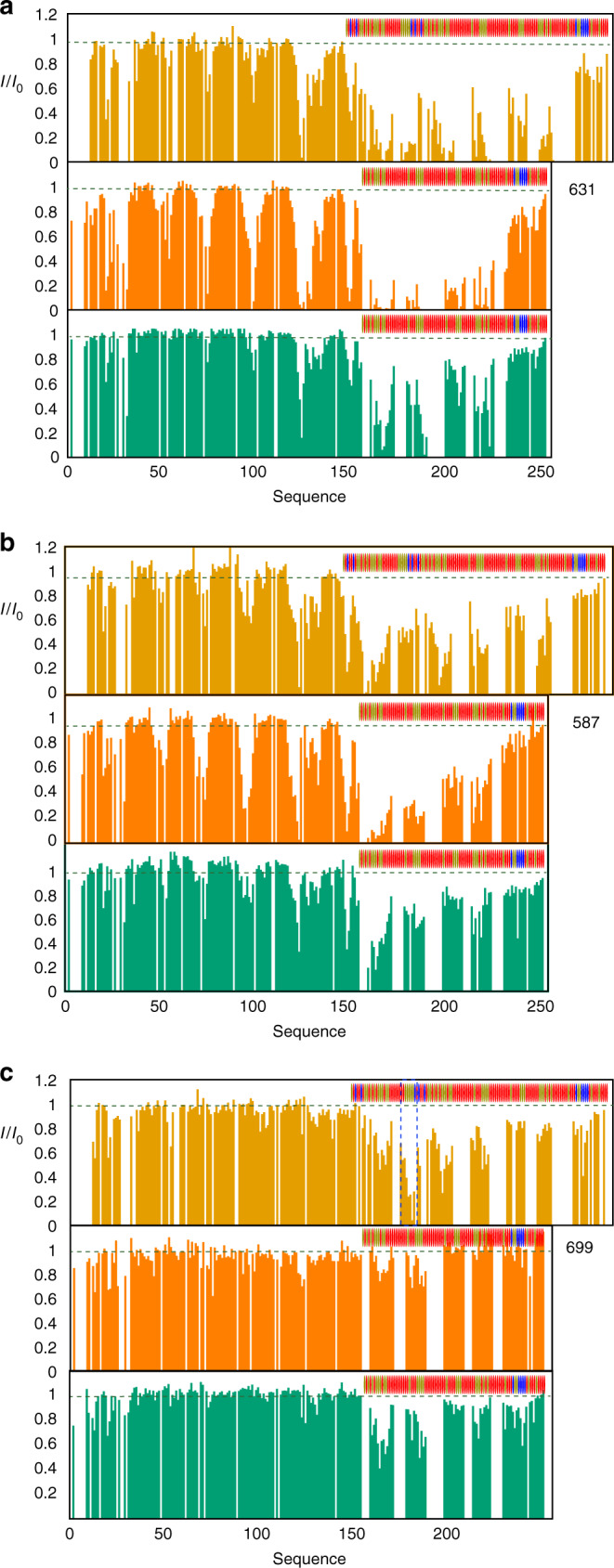
Adapted interaction modes are significantly weakened in the cross-interaction *h*ANP32A:627-NLS(E). Comparison of intermolecular contacts in *h*ANP32A:627-NLS(K) (light orange), *av*ANP32A:627-NLS(E) (dark orange) and *h*ANP32A:627-NLS(E) (green). Three PREs are compared, **a** 631, **b** 587 and **c** 699. The comparison of PRE profiles due to the presence of a spin probe on residue 631 illustrates the reduced number and strength of contacts of the IDD, with the 627 domain in the *h*ANP32A:627-NLS(E) complex compared to *h*ANP32A:627-NLS(K). Residue 587 shows a similar effect. Comparison of PRE profiles due to the presence of a spin probe on residue 699 illustrates the lack of interaction with the linker-NLS region in the *h*ANP32A:627-NLS(E) compared to *av*ANP32A:627-NLS(E). The position of the hexapeptide region is identified with dotted vertical lines in **c**.

Importantly, comparison of PRE profiles of *h*ANP32A:627-NLS(E) demonstrates that the cross-interaction, that represents the case encountered when a non-adapted avian IAV infects human cells, shows less extensive and in general weaker contacts compared to *h*ANP32A:627-NLS(K) and *av*ANP32A:627-NLS(E) (Fig. 5). The absence of K627 is again seen to diminish the numerous polyvalent contacts present in *h*ANP32A:627-NLS(K) as illustrated by the profiles induced by probes attached to 587 and 631, while the shorter IDD and lack of the hexapeptide abrogates the extensive interaction surface and the linker-specific contact present in *av*ANP32A:627-NLS(E) (compare profiles induced by probe attached at 699). The required components for interaction modes specific to either 627-NLS(K):*h*ANP32A or 627-NLS(E):*av*ANP32A are therefore both weakened when avian polymerase interacts with *h*ANP32A.

### *h*ANP32A:627-NLS in the context of integral FluB polymerase

**Fig. 6:**
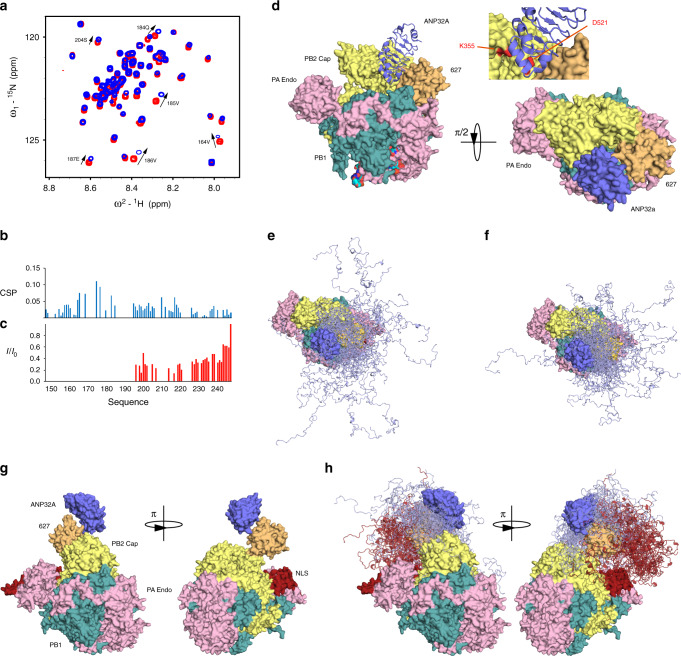
Interaction of hANP32A with full-length FluB polymerase. **a** Chemical shifts of ^15^N-labelled IDD of *h*ANP32A (4 μM) upon addition of full-length FluB polymerase (32 μM) bound to viral promoter RNA (*v*RNA). Measurement at 850 MHz, 293 K. Red—free protein, blue—in presence of polymerase. Notably the distribution of chemical shifts is highly similar to that induced in *h*ANP32A upon addition of IAV 627-NLS(K). **b** CSP associated with **a**. **c** Intensity ratio of ^15^N-labelled full-length *h*ANP32A (4 μM) upon addition of full-length FluB polymerase (32 μM) bound to *v*RNA. **d** Compatibility of the experimentally observed binding mode in the context of the vRNA-bound conformation of FluB polymerase^26^. The 627 domain was superimposed on the 627 domain of PB2 in the full-length polymerase structure (4wsa). In this position, ANP32A LRR can be accommodated in a large pocket formed by 627 (yellow–orange), and cap-binding domains of PB2 (yellow) and bordered by PB1 (dark-cyan). Inset: PB2 adaptive mutants D521 and K355 lie in the immediate vicinity of ANP32A LRR. **e** Conformational sampling of the IDD of *h*ANP32A, assuming the position of the LRR of ANP32A shown in **d**. The linker and NLS domains are not shown for clarity and are assumed flexible. **f** Conformational sampling of the IDD of *av*ANP32A (otherwise as in **e**). **g** Compatibility of the experimentally observed binding mode in the context of the cRNA-bound conformation of FluB^29^. The 627 domain was superimposed on the 627 domain of PB2 in the full-length polymerase structure (5epi), which is dislocated relative to the vRNA-bound form. ANP32A LRR can be accommodated easily on the accessible surface of 627. The NLS domain (brick red) is detached from 627 in this structure. **h** Conformational sampling of the IDD of *h*ANP32A, assuming the position of the LRR of ANP32A shown in **g**. Sampling of the IDD (light blue) linker and NLS domains are shown (brick red).

To determine whether the interactions characterised here are relevant in the context of a human-adapted full-length influenza polymerase, we initially repeated the chemical shift titrations using the heterotrimeric influenza B (FluB) polymerase (bound to *v*RNA), with the IDD of *h*ANP32A (Fig. 6a). The interaction sites are found to be the same as for 627-NLS from IAV (Fig. 6b). In the case of full-length *h*ANP32A (Fig. 6c), the first 50 amino acids of the IDD disappear from the spectrum, likely because the large particle results in extreme line broadening for the residues that are closest to the LRR domain due to slow tumbling in solution. Given the spectroscopic similarities of the interaction of ANP32A with IAV 627-NLS and full-length FluB polymerase, it is interesting to speculate whether the interaction described in detail for IAV 627-NLS can be accommodated within known structures of the polymerase.

Superposition of the 627 domains of the ensembles describing the complex onto the 627 domain in the transcription-active conformation of FluB polymerase (the form used in the interaction study described above)^26,32^ reveals that the LRR of ANP32A can indeed be inserted into a broad cylindrical pocket formed by 627, the mid and cap-binding domains of PB2, also bordered by the C-terminal domain (CTD) of PB1, allowing ANP32A to adopt the pose determined in solution (Fig. 6d). Interestingly, the site of recently described adaptive mutations of PB2 at 521 and 355^33^ lie in the immediate vicinity of the surface of ANP32A in this pose (inset Fig. 6d), suggesting that the importance of these mutations involves interaction with ANP32A. Figure 6e, f illustrates the expected conformational space sampled by the IDDs of *av* and *h*ANP32A within the 627-NLS:ANP32A complexes, indicating a broader capture radius for the avian complex. A similar procedure was applied to the conformationally very distinct transcriptionally inactive IAV polymerase bound to the *c*RNA 5′ terminus^29^, where the 627 domain sits on the surface, and is displaced and rotated relative to the polymerase core by ~70°^34^. In this case, the 627-NLS:ANP32A complex can be easily accommodated (Fig. 6g, h). The position of NLS on the surface of the polymerase, and its observed positional variability in existing structures, suggests that the open form of 627-NLS is sampled in both transcriptionally active and inactive polymerases. Finally, the recently determined dimeric structure of apo IAV polymerase accommodates the ensemble equally well (Supplementary Fig. 9)^35^.

## Discussion

In this study, we describe and compare the molecular complexes formed by the human-adapted or avian-adapted 627-NLS domains with the respective ANP32A host proteins, in order to understand the nature and specificity of these interactions. All of the implicated proteins exhibit extensive intrinsic disorder. The elaboration of atomic resolution descriptions of such highly disordered complexes requires methodologies that can account for the ensemble of structures sampled by the two proteins in solution^36,37^. Investigation of the *h*ANP32A:627-NLS(K) and *h*ANP32A:627-NLS(E) complexes using complementary structural data from NMR chemical shifts, spin relaxation and paramagnetic relaxation combined with quantitative ensemble modelling reveals the existence of highly dynamic molecular assemblies that exhibit very different interaction modes.

The LRR of ANP32A interfaces with the 627 domain of PB2 at the C-terminal end of its concave surface, apparently stabilised via hydrophobic and electrostatic interactions. This interaction is intrinsically very weak, but experimental NMR, including CSP and PREs, accurately report on the relative position of the two domains. The disordered domain interacts transiently with a basic patch on the surface of the 627 domain, preferentially in the case of *h*ANP32A:627-NLS(K), involving weak, polyvalent interactions of acidic and hydrophobic residues of the IDD. The presence of multiple low-affinity interactions (in the millimolar range individually) distributed throughout 80 amino acids of the IDD of *h*ANP32A results in an effective increase in affinity to ~50 μM for 627-NLS(K), an effect of avidity that has been observed in a number of systems exhibiting extensive intrinsic disorder^38,39^. The critical E627K mutation completes a continuous ridge of solvent-exposed positively charged residues^4^ that are available for interaction with the highly dynamic acidic IDD. This ridge colocalizes interacting residues of the IDD in the vicinity of the surface, that differentially implicates residues 589 and 591, 629, 631, 635 and 637 when the E627K mutation is present.

By contrast the IDD of *av*ANP32A interacting with 627-NLS(E) populates fewer conformations in the vicinity of the surface of 627. This lack of interaction, due to the interruption of the positively charged surface by the presence of E627, is compensated by an even broader conformational sampling of the IDD that exploits a more extensive interaction surface, implicating the hexapeptide motif specific to *av*ANP32A and the NLS domain, and in particular the linker region of 627-NLS. It is again interesting to note that two adaptative mutations (V683T and A684S)^40^ have been identified in this linker region. By binding predominantly to the open form of 627-NLS, ANP32A potentiates the equilibrium of open and closed forms of 627-NLS, an effect that is more efficient in the case of the 627-NLS(E):*av*ANP32A interaction, likely as a result of the interaction of the hexapeptide. Investigating the interaction of individual peptides isolated from the IDD, we are able to confirm the multivalent nature of the interaction of the *hu*IDD with 627-NLS, and to strongly substantiate the differential binding modes of the two complexes.

Notably, the ‘cross-interaction’ between *h*ANP32A and 627-NLS(E) exhibits neither the stabilisation properties mediated by the *av*IDD in the *av*ANP32A:627-NLS(E) complex, nor the polyvalent binding specific to 627(K) as observed in the *h*ANP32A:627-NLS(K) interaction. This lack of adapted molecular mechanisms results in fewer and weaker contacts between human ANP32A and avian-adapted 627-NLS. In combination, these effects may explain the inefficiency of avian polymerase in human cells in the absence of *av*ANP32A or 627-NLS(K).

It is interesting to compare the interaction results measured by different NMR-based techniques with existing studies, where results measured using different techniques seem to paint a slightly different picture. While the individual interaction sites reported in our study indicate a weaker binding between *av*ANP32A and 627-NLS(E) as compared to *h*ANP32A and 627-NLS(K), some biochemical and cell biology studies report on a higher affinity of *av*ANP32 to avian-adapted polymerase^17,24^. This apparent contradiction may result from different tagging techniques used in the reported pull-down assays, but we also note that overall affinities between proteins are not necessarily comparable to individual multivalent interactions in terms of affinities, and it will be interesting to resolve the dependencies in the future.

Importantly, NMR indicates that the complex characterised for the minimal 627-NLS:ANP32A interaction is maintained in the context of the integral FluB polymerase in its transcriptionally active form, showing very similar NMR-binding characteristics to *h*ANP32A. Superposition of the binding pose of ANP32A with respect to 627 onto the 627 domain in the associated transcriptionally active polymerase structure^26^ would place the LRR domain in a similarly dimensioned pocket bounded by PB2 domains (Fig. 6d). Intriguingly, recently characterised host-adaptive PB2 mutants^33^ lie in the immediate vicinity of the surface of the folded domain of ANP32A in this binding conformation. Influenza polymerase exhibits extensive plasticity in solution, as demonstrated by recent electron microscopy and X-ray crystallographic studies describing multiple states of the polymerase^26,28,29,34,35,41,42^, and it appears highly likely that many of these states will be in conformational exchange in solution. It is also possible that the highly dynamic and transient nature of the interaction with ANP32A enables or enhances exchange between these forms during the viral cycle. It is therefore of interest to investigate the possible impact of our observed binding mode on other known states of influenza polymerase. The proximity of ANP32A IDD to the interface between the 627 and mid-domains of PB2, both of which undergo large-scale reorientations and dislocation between apo-, *c*RNA-bound and *v*RNA-bound polymerase, also raises the possibility that ANP32A interaction is associated with these conformational changes. In the transcriptionally inactive structure, the 627 domain appears almost dislocated from the core, a conformation that can easily accommodate the binding pose of ANP32A determined for the minimal complex.

The information contained in our ensemble descriptions of the ANP32A:627-NLS interactions therefore allow us to speculate further on its role in viral function. Recent observations have established that dimerisation of influenza polymerase is essential for the initiation of *v*RNA synthesis during replication^35^. The apo-polymerase structure of IAV polymerase that was recently solved in its dimeric form is also found to be compatible with the binding pose determined here (Supplementary Fig. 9), again with the 627 domain dislocated and sitting on the surface of the catalytic core domains. It has been suggested that ANP32A plays a role in assembly or regulation of this dimerisation process, for example, by recruitment of a second apo ‘packaging’ polymerase to a replicating polymerase to initiate formation of the progeny viral RNP^43^. In this context, the polyvalent nature of the interaction between ANP32A and 627-NLS may be of functional relevance, allowing for more than one polymerase to simultaneously bind to ANP32A thereby colocalizing two polymerases to facilitate viral replication. In all of these aspects, the more extensive effective capture radius of the IDD of *av*ANP32A may be important.

It is known that the intrinsically disordered, phosphorylated CTD of host RNA polymerase II (Pol II) binds to the surface of PA of influenza polymerase to facilitate the cap-snatching mechanism^44,45^. Given the highly negatively charged nature of the IDD of ANP32A it seems possible that it may play a regulatory role in this interaction, for example, by competing with the phosphorylated CTD to inhibit the interaction with Pol II. Notably, the extremely long IDDs, allied to the fact that ANP32A is bound to the 627 domain that exhibits extensive mobility with respect to the rest of the polymerase core, would appear to facilitate the kind of flexible chaperoning action seen in other highly dynamic viral proteins^46^.

In summary, the description of these highly dynamic species-specific assemblies reveals unique mechanistic insight into the role of the ANP32 family in host adaptation of avian influenza polymerase to the human cells, and provides a molecular framework for understanding the considerable volume of experimental observation measured on this complex system, as well as informing the identification of novel targets for IAV inhibition.

## Methods

### Constructs

A codon optimised 627-NLS construct from PB2 subunit was synthesised encoding amino acids 538–759 from avian H5N1 A/duck/Shantou/4610/2003 for expression in *Escherichia coli* (Geneart, Regensburg, Germany)^27^. In addition, constructs containing just the 627 domain (amino acids 538–693) or the NLS domain (amino acids 678–759) were generated. Avian ANP32A (*Gallus gallus*, XP_413932.3) was synthesised and codon optimised for expression in *E. coli* (GenScript, New Jersey, USA). Plasmids containing just the intrinsically disordered region of avian ANP32A (avIDD, amino acids 149–281) or human ANP32A (hIDD, amino acids 144–249) were generated. All constructs were cloned into a plasmid derived from pET9a with an N-terminal His-tag and a TEV cleavage site (MGHHHHHHDYDIPTTENLYFQG). pQTEV-ANP32A was a gift from Konrad Buessow (Addgene plasmid # 31563; http://n2t.net/addgene:31563; RRID:Addgene_31563)^47^.

### Protein expression and purification

Plasmids were transformed into *E. coli* Rosetta cells, and the cultures were grown in LB and induced with IPTG for 16 h at 18 °C. Bacteria were harvested by centrifugation, resuspended in buffer A (50 mM Tris-HCl pH 7.5 and 200 mM NaCl) with protease inhibitors (complete, Roche) and bacterial lysis was performed by sonication. All proteins were purified by affinity chromatography on Ni-NTA agarose (Qiagen), followed by incubation with TEV protease at 4 °C coupled with dialysis into buffer A. A second affinity column with Ni-NTA agarose was performed and the flow-through was loaded into a Superdex 75 column (GE Healthcare) for size-exclusion chromatography in buffer A.

To produce ^15^N-labelled or ^15^N, and ^13^C-labelled proteins for NMR spectroscopy, bacteria were grown in M9 minimal medium containing MEM vitamins (Gibco), supplemented with 1 g L^−1^ of ^15^NH_4_Cl and 2 g L^−1^ of unlabelled or ^13^C-glucose. To produce additionally ^2^H-labelled proteins, the M9 minimal medium was prepared in D_2_O and 2 g L^−1^ of deuterated ^13^C-glucose. Protein purity was checked by SDS–PAGE and mass spectrometry. Single-point mutations of 627-NLS were done using the Quick change method^48^, using Phusion high-fidelity DNA polymerase and DpnI (Thermo Scientific). Cysteine mutants were purified as mentioned above for wild-type protein; however, 10 mM of dithiothreitol (DTT) was added after the second Ni-NTA column to keep proteins in a reduced state until labelling. The heterotrimeric human influenza polymerase from B/Memphis/13/03 (FluB) was expressed as a self-cleaving polyprotein and purified, using NTA affinity and heparin columns followed by size-exclusion chromatography^32^. ANP32A peptides were purchased from Caslo, Denmark.

### NMR spectroscopy

All samples for NMR were measured in 50 mM Tris-HCl buffer pH 6.5, 200 mM NaCl and 10% D_2_O. The assignment of the intrinsically disordered regions of avian and human ANP32A were obtained using ^15^N,^13^C-labelled samples (700 μM) using BEST-TROSY tridimensional experiments recorded on a Bruker spectrometer equipped with a cryoprobe operating at 20 °C and a ^1^H frequency of 850 MHz. All spectra were processed using NMRPipe^49^ and analysed in Sparky^50^. MARS^51^ was used for spin system identification, followed by manual verification. The folded domain of ANP32A has been assigned previously^52,53^. ^13^C^α^ chemical shifts of the intrinsically disordered regions were compared to random coil values using the software SSP^54^.

^15^N R_1ρ_ relaxation rates were measured at 293 K and a ^1^H frequency of 850 MHz using a spin lock of 1.5 kHz as described^55^. A typical set of relaxation delays included points measured at 1, 15, 30, 50, 100, 140, 200 and 230 ms, including repetition of one delay. Relaxation rates were determined using in-house software and errors were estimated on the basis of noise-based Monte Carlo simulation. Interaction experiments with full-length polymerase were acquired with ^15^N-labelled *h*IDD or full-length ^15^N-*h*ANP32A at a concentration of 4 μM after the addition of 32 μM of human FluB polymerase bound to the 5′ terminal viral RNA promoter (5′-pAGUAGUAACAAGAG-3′ OH). These experiments were recorded at 293 K and a ^1^H frequency of 850 MHz.

PRE effects used to model the complex formed by ANP32A (^15^N labelled, human or avian) and 627-NLS (E627 or K627) were measured from the peak intensity ratios between a ^15^N-HSQC 2D spectrum recorded on a sample containing 627-NLS labelled with TEMPO, and a reference diamagnetic sample that was incubated previously with 5 mM of DTT. For these experiments, single-cysteine mutants at positions 539, 587, 605, 631, 643, 699, 707 and 717 were tagged using 4-maleimido-TEMPO. Briefly, purified 627-NLS single-cysteine mutants were reduced with 10 mM of DTT at 4 °C for 12 h and then dialysed throughly into 50 mM phosphate buffer pH 7.0 containing 150 mM NaCl without DTT. A fivefold molar excess of 4-maleimido-TEMPO dissolved in DMSO was added to the reduced 627-NLS cysteine mutants. The reaction was incubated for 12 h at 4 °C and then injected into a Superdex S75 column to eliminate the excess of TEMPO through size-exclusion chromatography. Complete labelling with TEMPO was verified by mass spectrometry. Measurement of PRE effects was performed in samples containing 200 μM of ^15^N-labelled hIDD, and 100 or 200 mM of the respective TEMPO-labelled 627-NLS (K627) mutants. Measurements in full-length *h*ANP32A were performed with 300 μM of ^15^N-*h*ANP32A and 150 μM of the 627-NLS (K627) mutants, and measurements on full-length *av*ANP32A were carried out with 220 μM of ^15^N-labelled protein and 400 μM of the 627-NLS (E627) mutants.

### Determination of the relative position of ANP32A and 627

Experimentally determined PREs measured on *h* and *av*ANP32A in the presence of different spin-labelled forms of *h* and *av*627-NLS, respectively, were used to determine the relative position of the folded domain of ANP32A with respect to the 627 domain. Two thousand different positions of the two domains were generated using the programme Haddock^56^, varying over a wide range of distances and orientations. Positions of spin-label-bearing sidechains were generated on the basis of rotameric libraries (see Supplementary Fig. 8)^31,37^, and an ensemble of sidechain positions was used to calculate expected PREs on ANP32A for a given position of the 627 domain for each label. Admixtures were adjusted to ensure a population of the bound state of 10% for both complexes. The position of each of the 2000 starting conformations were varied over a range of ±10 Å along three orthogonal cartesian axes at a resolution of 0.1 Å, and the best fitting position retained. The ten best fitting structures are shown in Fig. 3d.

### Ensemble descriptions of ANP32A:627-NLS complexes

Having determined the relative position of the two domains, the flexible parts were constructed onto this conformation. For both *h* and *av* complexes, the statistical coil model flexible meccano^57^ was used to predict 10,000 conformations of the linker region of 627-NLS, the NLS domain, the NLS peptide that terminates 627-NLS, and the 96 or 128 amino acid IDD of *h* and *av*ANP32A. Conformers were calculated using amino acid-specific potentials that reproduce the experimentally observed behaviour of the IDD domains, and were calculated to avoid steric overlap between any of the domains (Supplementary Fig. 8). PREs over the entire ANP32A molecule (folded and unfolded domains) were calculated for each of the conformers calculated for each complex, and these conformations were used as a basis set from which ensembles were selected using the ASTEROIDS approach^36,58^. Ensemble size was estimated on the basis of direct and cross-validated PRE profiles (60 conformers were used for both *h* and *av* complexes).

Distance matrices were calculated by calculating the average distance between C^α^ atoms between the two proteins in the selected ensembles from the two complexes, and the distance difference matrix shown in Fig. 5a by subtracting the matrix from *h*ANP32A:627-NLS(K) from the *av*ANP32A:627-NLS(E) matrix.

### Reporting summary

Further information on research design is available in the Nature Research Reporting Summary linked to this article.

## Supplementary information

Supplementary Information

Reporting Summary

## Data Availability

All of the NMR data presented in the article are available from the authors upon request. The NMR chemical shift assignments, and PREs of avian and human ANP32A have been deposited in the Biological Magnetic Resonance Bank (BMRB; bmrb.wisc.edu/) with accession codes 28134 and 28135, respectively.
