# Supplementary material for: Molecular basis of host-adaptation interactions between influenza virus polymerase PB2 subunit and ANP32A
Source: Nat Commun. 2020 Jul 21;11:3656. doi: 10.1038/s41467-020-17407-x (PMC7374565; doi:10.1038/s41467-020-17407-x)
Supplement: Supplementary file 1 — Supplementary Information [file 41467_2020_17407_MOESM1_ESM.pdf]

Supplementary Information

Molecular Basis of Host-Adaptation Interactions between Influenza Virus Polymerase PB2 and ANP32a

Camacho Zarco et al

Supplementary Table 1

Affinities of different complexes

Residue	Kd (μM)[a]	Std Error
	**^{15}N -627E + avIDD**	
651	630.58	218.8
652	743.08	257.6
662	994.59	229.4
682	871.17	224.2
	**^{15}N -627K + *h*IDD**	
588	16.22	1.99
591	20.19	4.04
645	17.57	3.16
651	20.22	2.99
652	30.35	7.44
681	34.3	12.7
682	36.74	12.06
	**^{15}N -NLS + *av*IDD**	
688	457.63	11.9
690	430.8	25.6
738	522.3	41.8
739	400.82	39.15
	**^{15}N -NLS + *h*IDD**	
688	605.04	55.63
690	602.36	23.19
738	583.44	50.56
739	547.73	84.14
	**^{15}N -*h*IDD + 627E**	
178	2350.4	641.7
183	1699.9	191.2
184	1436.6	230.3
185	1898.5	109
186	1797.2	123.1
	**^{15}N -*h*IDD + 627K**	
183	779.3	91
184	817.1	81.4
185	965.3	88.7
186	1008.3	59.1

187	867.1	104.9
	**^{15}N -*av*IDD + 627E**	
217	1730.2	335.4
218	1884.5	294.2
219	1807.4	314.7
220	1848.0	274.3
	**^{15}N -*h*LRR + 627-NLS (K)**	
119	1575	82.9
120	1700	100.2
121	1670	58
122	1659	61
	**^{2}H,^{15}N -*h*ANP32A + 627NLS(K)**	
98	1581	59
122	1407	79.7
	**^{2}H,^{15}N -*av*ANP32A + 627NLS(E)**	
122	>3000	
	**^{2}H,^{15}N-627NLS(K) + *h*ANP32A**	
644o	28.9	6.44
645o	42.2	3.6

A – All K_Ds were estimated using NMR spectroscopy via chemical shift titrations (see figure S3)

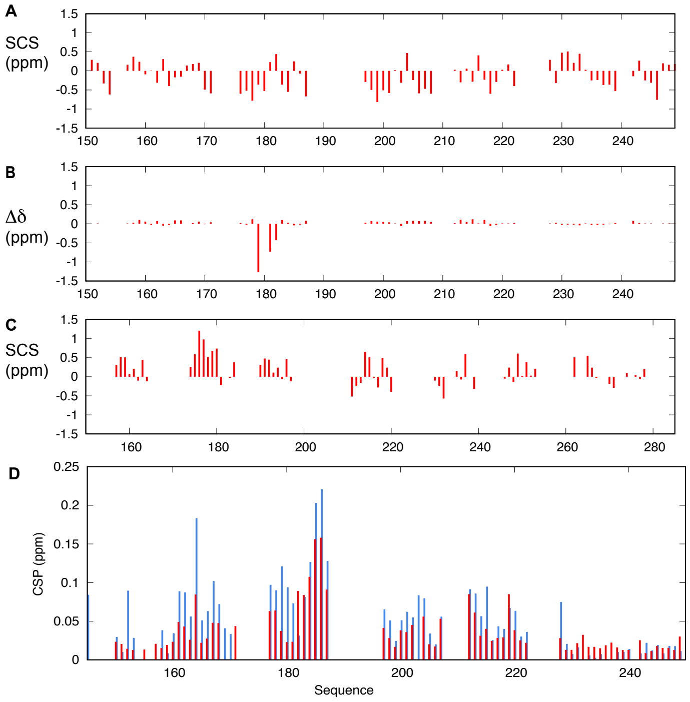

Supplementary Figure 1. ANP32a C-terminal domains adopt intrinsically disordered conformations in solution.

A. Secondary $^{13}C^{\alpha}$ chemical shifts along the sequence of *h*ANP32a IDD. Values are all close to zero, indicating negligible propensity for secondary structure in solution.

B. $^{13}C^{\alpha}$ chemical shift perturbation (CSP) of *h*ANP32a IDD upon interaction with *h*627-NLS. Chemical shifts were determined from heteronuclear 3D BEST-TROSY three dimensional experiments. Only the ^{179}YDED^{182} region shows evidence of conformational change upon binding.

C. Secondary $^{13}C^{\alpha}$ chemical shifts along the sequence of *av*ANP32a IDD. Values are again close to zero, indicating negligible propensity for secondary structure in solution except for the hexapeptide ^{176}VLSLVK^{181} that shows weak (approximately 20%) helical propensity.

D. Comparison of ^{15}N, ^{1}H chemical shift perturbation between *h*ANP32a IDD:627K (red bars) and *h*ANP32a:*h*627-NLS (blue bars). *h*ANP32a IDD:627K spectra were recorded at 293K with concentrations of 300 μM (1:1 mixture). *h*ANP32a: *h*627-NLS spectra were recorded at 293K, with concentrations of 25 and 50μM respectively.

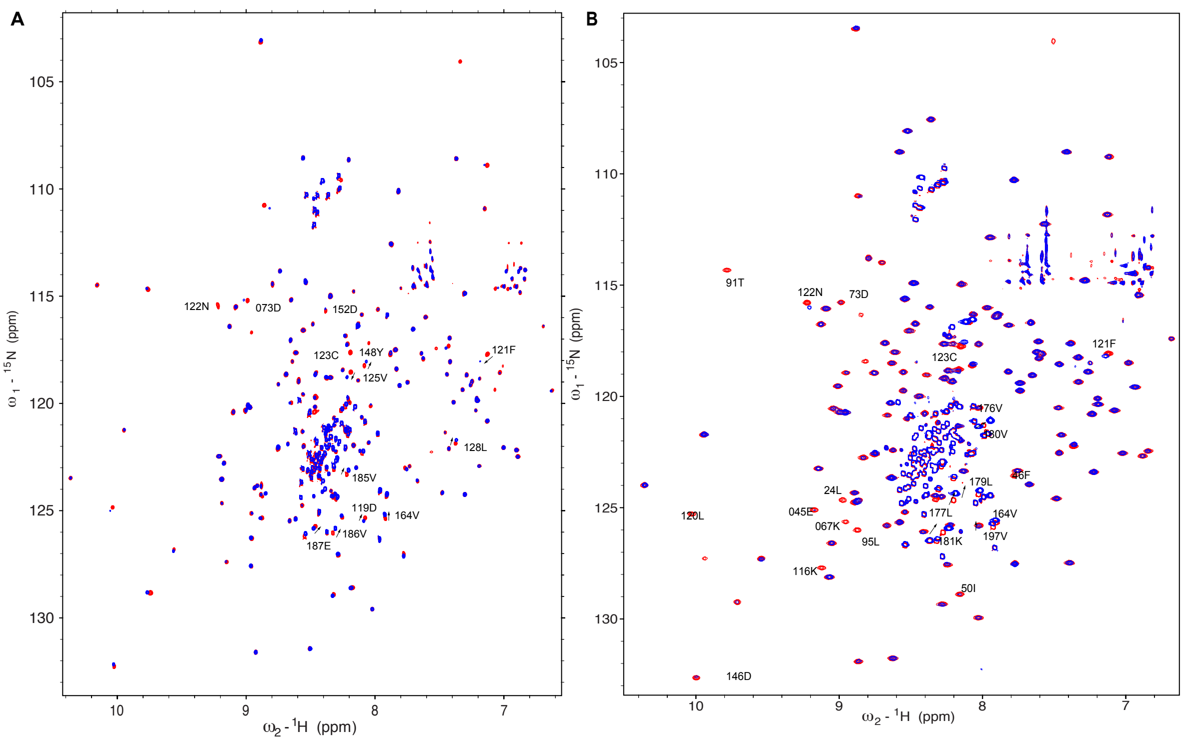

Supplementary Figure 2.

A. HSQC of (red) ^{15}N labelled *av*ANP32a (300μM) upon addition of *av*627-NLS (600μM) (blue). Data were recorded at 950MHz, 293K.

B. TROSY of (red) ^{15}N labelled *av*ANP32a (300μM) upon addition of *av*627-NLS (600μM) (blue). Data were recorded at 700MHz, 293K.

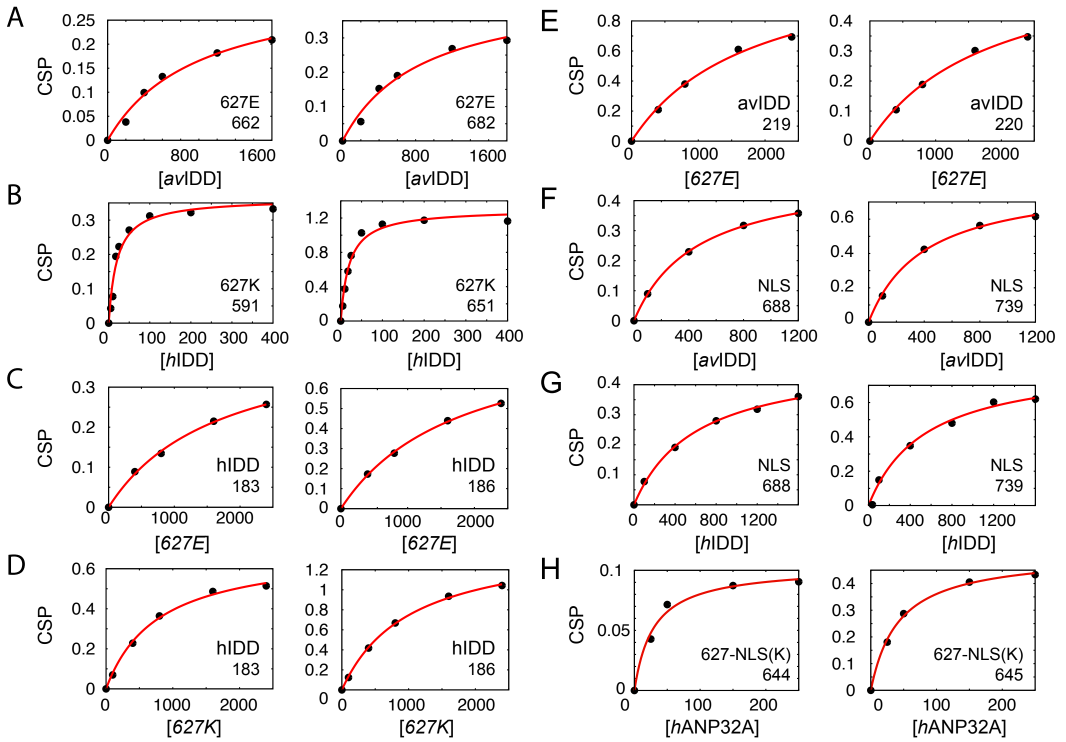

Supplementary Figure 3. Examples of chemical shift titrations associated with K_D values estimated in table S1.

Concentrations of the titrated partner are shown on the x-axis in µM. The residue number is given for each peak titration. Concentrations of the observed, labelled proteins were: A) 200 µM of ^{15}N-627E, B) 200 µM of ^{15}N,^{13}C-627K, C) 200 µM of ^{15}N-hIDD, D) 200 µM of ^{15}N-hIDD, E) 200 µM of ^{15}N,^{13}C-avIDD, F) 180 µM of ^{15}N-NLS, G) 180 µM of ^{15}N-NLS and H) 250 µM of ^{2}H,^{15}N-627-NLS(K).

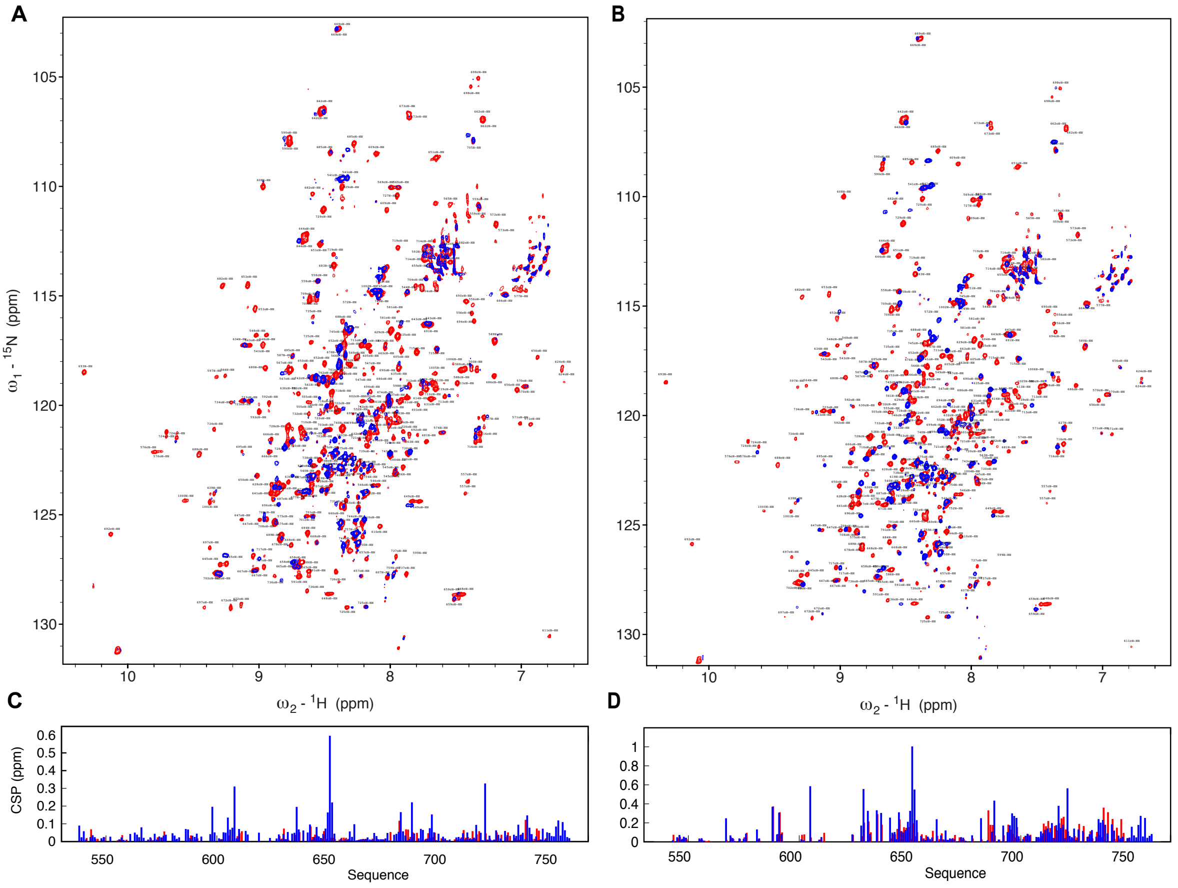

Supplementary Figure 4.

A. TROSY spectra of ^{2}D, ^{15}N labelled *h*627-NLS alone (red) (250μM) and in the presence of unlabelled of *h*ANP32a (250μM). Spectra acquired at 950MHz and 293K.

B. TROSY spectra of ^{2}D, ^{15}N labelled *av*627-NLS alone (red) (250μM) and in the presence of unlabelled of *av*ANP32a (250μM). Spectra acquired at 950MHz and 293K.

C. Chemical shift perturbation (CSP) of *av*627-NLS (250 μM) upon addition of full length *av*ANP32a at a ratio of 1:1. Blue: resonances corresponding to the open form. Red: resonances corresponding to the closed form.

D. Chemical shift perturbation (CSP) of *h*627-NLS (250 μM) upon addition of full length *h*ANP32a at a ratio of 1:1. Blue: resonances corresponding to the open form. Red: resonances corresponding to the closed form. All spectra were recorded on ^{2}D, ^{13}C, ^{15}N labelled 627-NLS at 293K and 850MHz.

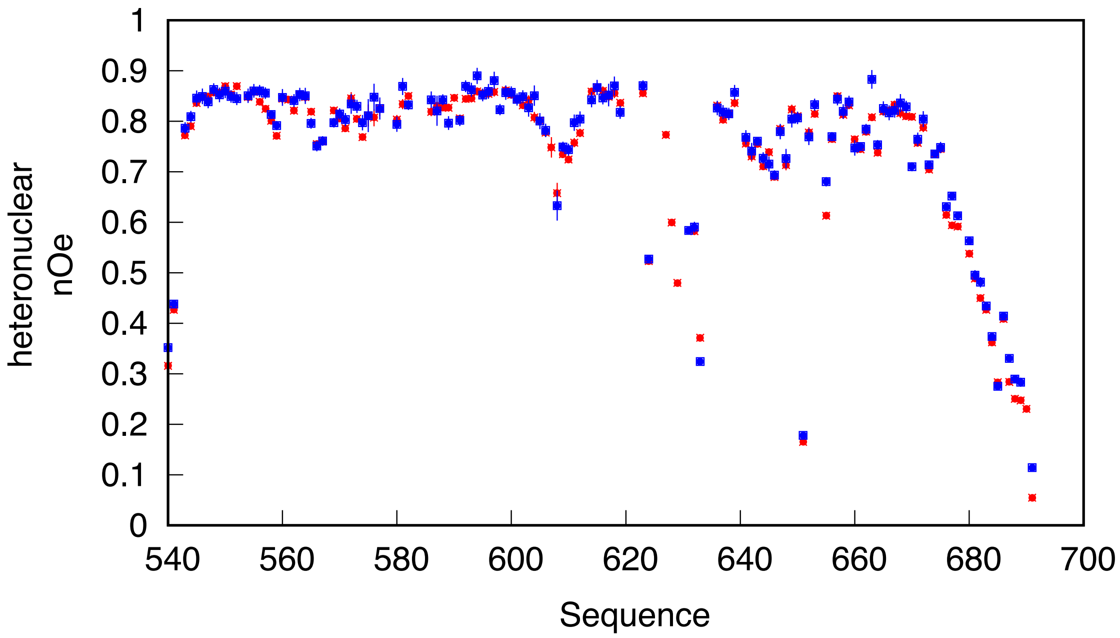

Supplementary Figure 5. Heteronuclear nOe of 627K (red) and 627E (blue) HSQC. Data were recorded at 850MHz, 298 K.

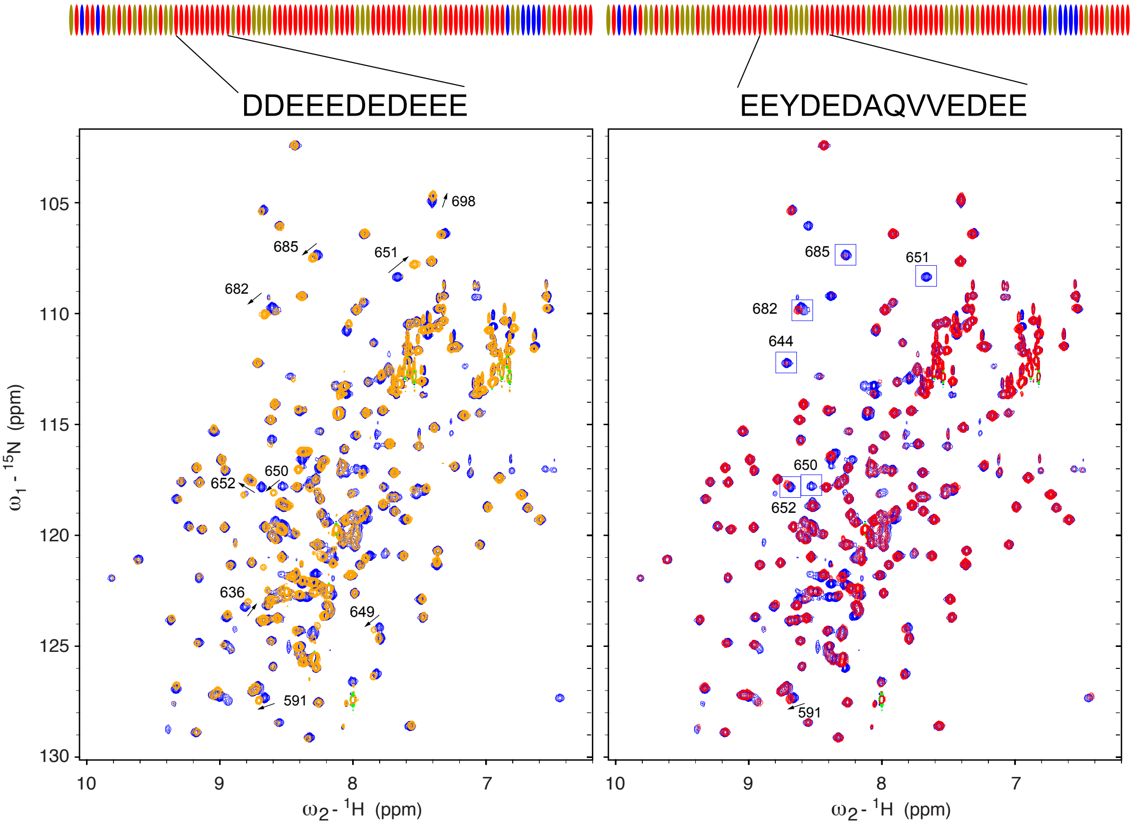

Supplementary Figure 6. Chemical shift perturbations of two peptides representing adjacent strands of *hu*ANP32A. HSQC spectra of the open-only form of ^{2}D, ^{13}C, ^{15}N labelled 627-NLS(K) (D730A/E687A) (200μM) (blue) indicating chemical shifts of selected sites upon addition of the indicated peptides (orange and red). Experiments were measured at 850 MHz and 293K.

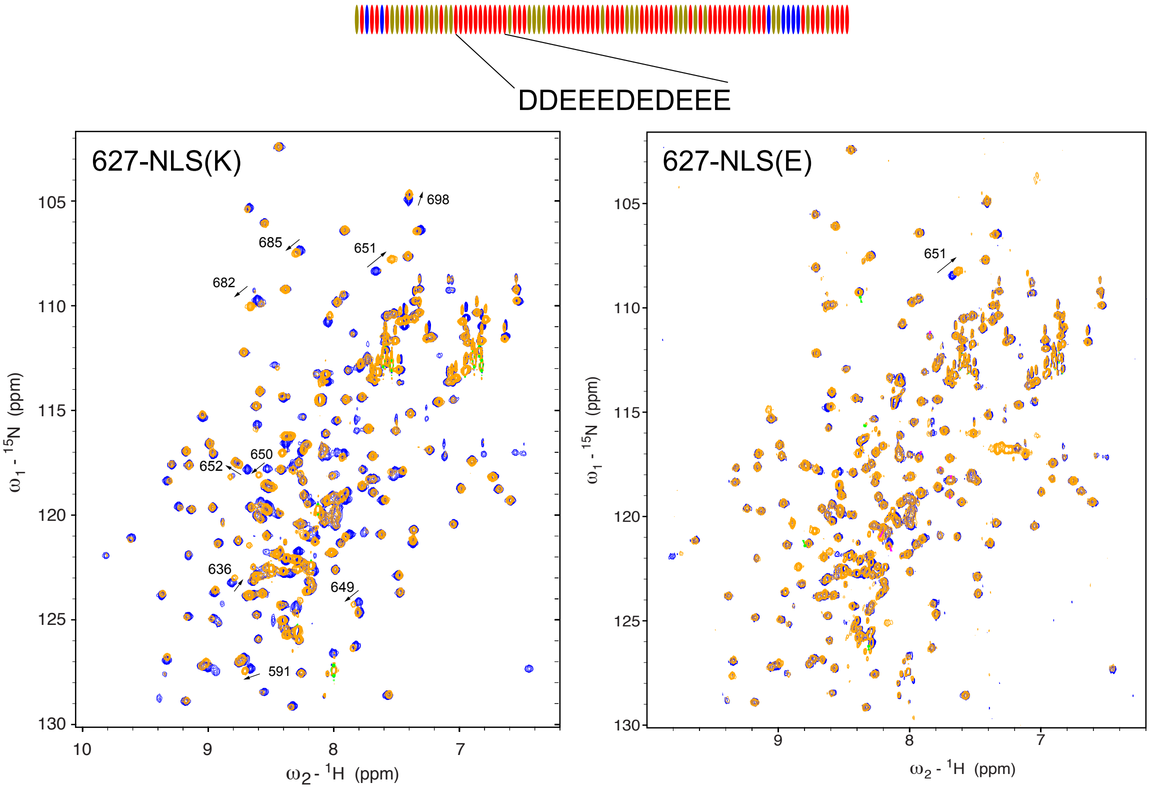

Supplementary Figure 7. Chemical shift perturbation of a negatively charged peptide representing a region of *h*ANP32A that occurs almost identically in *av*ANP32A (one D:E mutation). HSQC of the open-only form of ^{2}D, ^{13}C, ^{15}N labelled 627-NLS(K) (D730A/E687A) (200μM) (blue) indicating chemical shifts of selected sites upon addition of the indicated peptides (orange). Experiments were measured at 850 MHz and 293K. The peptide concentration for the left hand (627-NLS(K)) spectrum was 250μM compared to 1.5mM for the right-hand (627-NLS(E)) spectrum (at 250 μM no shifts are observed because the interaction affinity is too weak to be detectable at this concentration), highlighting the essential nature of K627 for the strength of this interaction. Experiments were measured at 850 MHz and 293K.

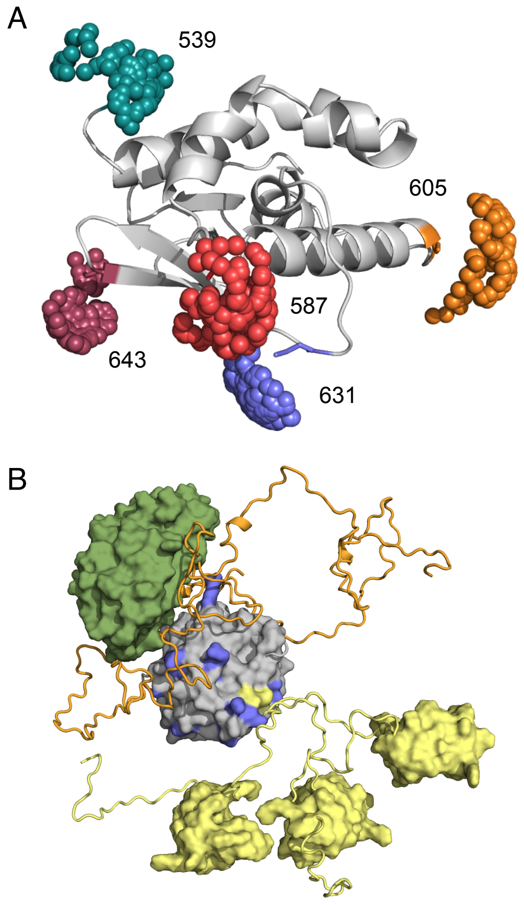

Supplementary Figure 8

A - Representation of the sampling of TEMPO maleimide side-chain with respect to the back conformation of 627. Similar calculations were made for the three spin-label sites on the NLS domain (not shown). Rotamer-specific libraries were randomly sampled to place the nitroxide group in available conformations that respect steric clashes with the remainder of the molecule for each of the conformations sampled in the pool of conformations.

B - Representation of the generation of conformers present in the pool of 10000 conformers generated using the flexible-meccano algorithm (three are shown here). The statistical coil model was used to randomly sample the conformational space available to the linker region between the 627 (grey) and NLS (yellow) domains, the NLS peptide constituting the C-terminus of the 627-NLS domains, and the IDD domain of ANP32a (orange). The folded domain of ANP32a as shown in surface representation (green).

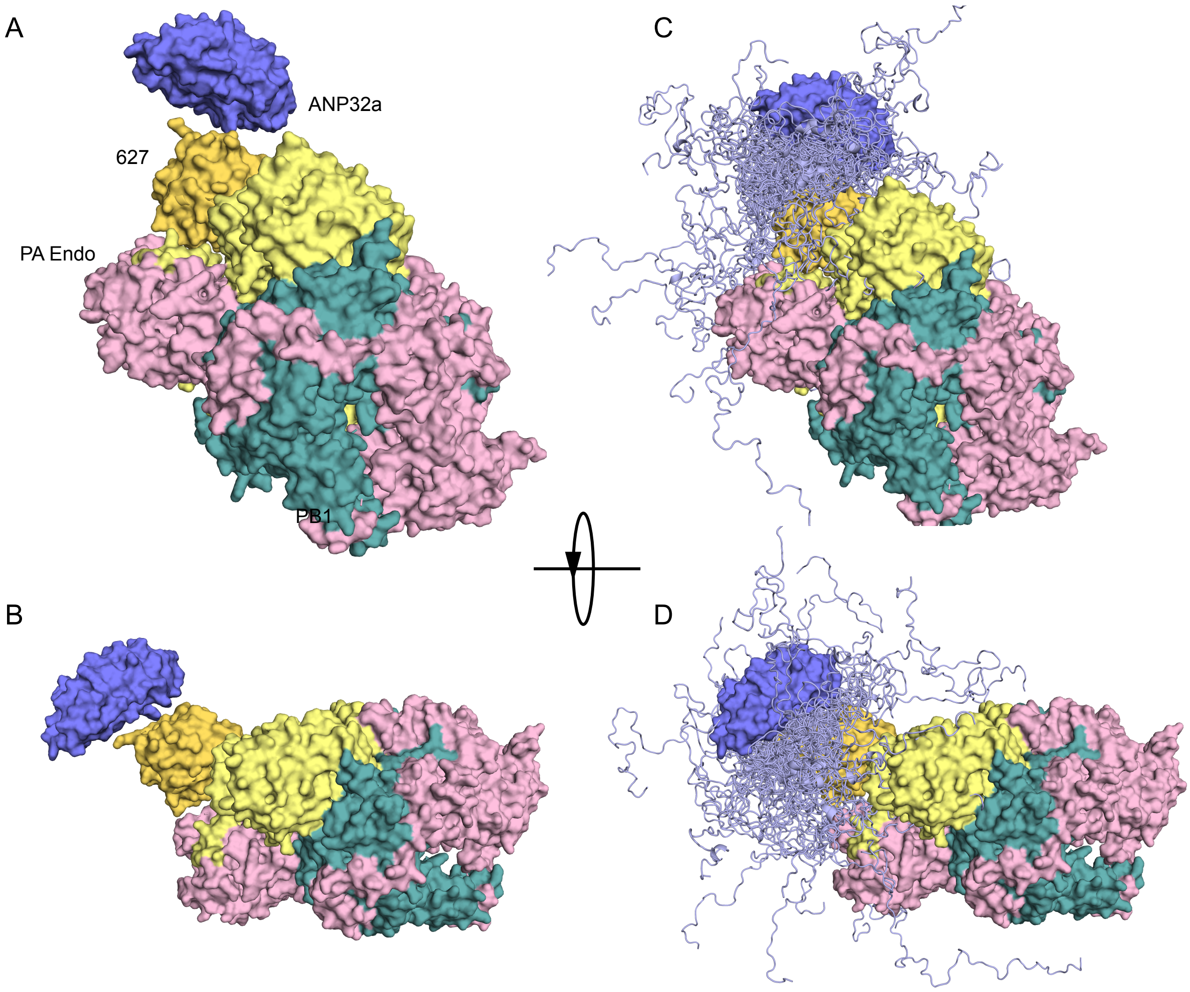

Supplementary Figure 9.

Compatibility of binding mode determined in free solution in the context of the apo conformation of influenza A.[1] A-B. The 627 domain was superimposed on the 627 domain of PB2 in the full length polymerase structure (6qnw). In this position, ANP32a folded domain can be adjacent to 627 (yellow-orange) on the surface of the polymerase. C-D. Conformational sampling of the IDD of *h*ANP32a, assuming the position of the folded domain of ANP32a shown in A and B. The linker and NLS domains are not shown for clarity and are assumed flexible.

REFERENCES

1. Fan, H. *et al.* Structures of influenza A virus RNA polymerase offer insight into viral genome replication. *Nature* **573**, 287–290 (2019).
